# Supplementary material for: Evaluation of renal injury and function biomarkers, including symmetric dimethylarginine (SDMA), in the rat passive Heymann nephritis (PHN) model
Source: PLoS One. 2022 May 27;17(5):e0269085. doi: 10.1371/journal.pone.0269085 (PMC9140233; doi:10.1371/journal.pone.0269085)
Supplement: S1 File — (DOCX) [file pone.0269085.s001.docx]

**Supporting Table 1.** Body weight data by group and time point in vehicle control and anti-Fx1A-treated rats.

Data presented as Mean ± SD, N = animals/group.

***** Anti-Fx1A group is significantly different (p ≤ 0.05) from vehicle control group at the same time point.

| **Treatment Group** | **Vehicle** | | | | | | **Anti-Fx1A 7.5 mL/kg** | | | | | |
| --- | --- | --- | --- | --- | --- | --- | --- | --- | --- | --- | --- | --- |
| **Sample Day** | **Predose** | **Dosing Phase** | | | | | **Predose** | **Dosing Phase** | | | | |
|  | Day 5 | Day 1 | Day 9 | Day 16 | Day 21 | Day 28 | Day 5 | Day 1 | Day 9 | Day 16 | Day 21 | Day 28 |
| **Body weight (g)** | 279.6 ± 9.2 | 290.9 ± 10.6 | 299.5 ± 12.8 | 339.8 ± 19.9 | 364.9 ± 26.5 | 397.0 ± 27.8 | 279.4 ± 9.2 | 292.0 ± 10.4 | 303.1 ± 15.1 | 335.4 ± 22.6 | 353.2 ± 27.4 | 364.7 ± 17.2* |
| **N** | 48 | 48 | 12 | 12 | 12 | 12 | 48 | 48 | 12 | 12 | 12 | 12 |

**Supporting Table 2.** Complete blood count data by group and time point in vehicle control and anti-Fx1A-treated rats.

| **Treatment**  **Group** | **Vehicle** | | | | **Anti-Fx1A 7.5 mL/kg** | | | |
| --- | --- | --- | --- | --- | --- | --- | --- | --- |
| **Sample Day**  **Post-treatment** | Day 9 | Day 16 | Day 21 | Day 28 | Day 9 | Day 16 | Day 21 | Day 28 |
| **Erythrocyte count**  **(cells X 10^6^/µL)** | 7.82 ± 0.43 | 8.06 ± 0.31 | 8.04 ± 0.29 | 8.36 ± 0.31 | 7.64 ± 0.36 | 7.91 ± 0.28 | 7.90 ± 0.48 | 7.80 ± 0.54* |
| **Hemoglobin**  **(g/dL)** | 14.9 ± 0.7 | 14.9 ± 0.7 | 14.9 ± 0.5 | 15.1 ± 0.7 | 14.5 ± 0.5 | 14.5 ± 0.4 | 14.3 ± 1.0 | 13.9 ± 1.2* |
| **Hematocrit**  **(%)** | 47 ± 2 | 48 ± 2 | 48 ± 1 | 48 ± 2 | 45 ± 2* | 46 ± 1* | 46 ± 3* | 44 ± 4* |
| **MCV**  **(fL)** | 59.6 ± 1.7 | 59.9 ± 2.0 | 59.7 ± 1.5 | 57.8 ± 1.7 | 58.4 ± 1.6 | 58.8 ± 1.6 | 58.1 ± 1.7* | 57.0 ± 1.8 |
| **MCH**  **(pg)** | 19.0 ± 0.7 | 18.5 ± 0.7 | 18.5 ± 0.5 | 18.1 ± 0.7 | 19.0 ± 0.6 | 18.3 ± 0.6 | 18.0 ± 0.6* | 17.9 ± 0.8 |
| **MCHC**  **(%)** | 31.9 ± 0.6 | 30.9 ± 0.5 | 31.0 ± 0.6 | 31.3 ± 0.5 | 32.5 ± 0.6* | 31.2 ± 0.4 | 31.1 ± 0.4 | 31.4 ± 0.5 |
| **Reticulocyte count**  **(cells X 10^3^/µL)** | 330.9 ± 38.8 | 319.3 ± 59.0 | 266.4 ± 41.1 | 261.2 ± 37.0 | 415.6 ± 67.0* | 290.2 ± 48.0 | 254.0 ± 64.1 | 340.1 ± 90.9* |
| **Total leukocyte count**  **(cells X 10^3^/µL)** | 8.93 ± 1.13 | 9.53 ± 2.64 | 9.77 ± 3.53 | 11.68 ± 2.85 | 10.26 ± 2.63 | 11.77 ± 3.64 | 10.45 ± 2.76 | 12.55 ± 3.03 |
| **Neutrophil count**  **(cells X 10^3^/µL)** | 1.84 ± 0.64 | 1.56 ± 0.45 | 1.55 ± 0.72 | 1.76 ± 0.41 | 2.13 ± 0.73 | 2.18 ± 0.48* | 1.93 ± 0.48 | 2.39 ± 0.63* |
| **Lymphocyte count**  **(cells X 10^3^/µL)** | 6.57 ± 0.93 | 7.52 ± 2.38 | 7.75 ± 2.74 | 9.35 ± 2.52 | 7.51 ± 1.97 | 9.02 ± 3.26 | 7.95 ± 2.27 | 9.53 ± 2.78 |
| **Monocyte count**  **(cells X 10^3^/µL)** | 0.29 ± 0.13 | 0.25 ± 0.11 | 0.25 ± 0.10 | 0.29 ± 0.11 | 0.35 ± 0.14 | 0.32 ± 0.12 | 0.33 ± 0.13 | 0.35 ± 0.12 |
| **Eosinophil count**  **(cells X 10^3^/µL)** | 0.15 ± 0.12 | 0.10 ± 0.04 | 0.09 ± 0.05 | 0.12 ± 0.03 | 0.15 ± 0.03 | 0.13 ± 0.08 | 0.11 ± 0.04 | 0.11 ± 0.05 |
| **Basophil count**  **(cells X 10^3^/µL)** | 0.03 ± 0.01 | 0.03 ± 0.02 | 0.02 ± 0.01 | 0.05 ± 0.05 | 0.03 ± 0.02 | 0.05 ± 0.03 | 0.03 ± 0.01 | 0.04 ± 0.02 |
| **Large unstained cells**  **(cells X 10^3^/µL)** | 0.07 ± 0.04 | 0.07 ± 0.06 | 0.10 ± 0.06 | 0.11 ± 0.04 | 0.08 ± 0.03 | 0.08 ± 0.04 | 0.10 ± 0.06 | 0.14 ± 0.07 |
| **Platelet count**  **(cells X 10^3^/µL)** | 899 ± 251 | 1018 ± 142 | 858 ± 205 | 952 ± 149 | 1218 ± 276* | 1369 ± 173* | 1110 ± 171* | 1222 ± 179* |

Data presented as Mean ± SD, N = 12/group.

***** Anti-Fx1A group is significantly different (p ≤ 0.05) from vehicle control group at the same time point.

**Supporting Table 3.** Clinical chemistry data by group and time point in vehicle control and anti-Fx1A-treated rats.

| **Treatment**  **group** | **Vehicle** | | | | **Anti-Fx1A 7.5 mL/kg** | | | |
| --- | --- | --- | --- | --- | --- | --- | --- | --- |
| **Sample Day**  **Post-treatment** | Day 9 | Day 16 | Day 21 | Day 28 | Day 9 | Day 16 | Day 21 | Day 28 |
| **Alanine aminotransferase**  **(IU/L)** | 34.3 ± 5.3 | 37.8 ± 5.2 | 34.5 ± 4.0 | 33.4 ± 5.4 | 36.2 ± 6.5 | 41.7 ± 8.3 | 34.9 ± 4.5 | 34.8 ± 4.6 |
| **Albumin**  **(g/dL)** | 4.1 ± 0.1 | 3.9 ± 0.2 | 4.1 ± 0.1 | 4.2 ± 0.2 | 3.6 ± 0.2* | 3.1 ± 0.5* | 3.2 ± 0.4* | 3.2 ± 0.4* |
| **Albumin/Globulin Ratio** | 2.2 ± 0.2 | 2.1 ± 0.2 | 2.2 ± 0.2 | 2.0 ± 0.2 | 1.9 ± 0.2* | 1.3 ± 0.5* | 1.3 ± 0.3* | 1.2 ± 0.3* |
| **Alkaline phosphatase (IU/L)** | 166.8 ± 30.6 | 169.9 ± 32.4 | 139.0 ± 16.3 | 120.7 ± 19.5 | 173.4 ± 34.9 | 146.5 ± 26.7 | 119.2 ± 18.8* | 97.3 ± 21.4* |
| **Aspartate aminotransferase (IU/L)** | 95.8 ± 26.0 | 95.9 ± 14.1 | 85.1 ± 13.2 | 91.1 ± 25.2 | 85.5 ± 14.3 | 83.9 ± 26.5 | 70.3 ± 14.3* | 73.8 ± 10.2* |
| **Calcium**  **(mg/dL)** | 10.6 ± 0.3 | 10.2 ± 0.5 | 10.3 ± 0.2 | 10.4 ± 0.2 | 10.4 ± 0.3* | 10.1 ± 0.3 | 10.3 ± 0.3 | 10.2 ± 0.3* |
| **Chloride**  **(mmol/L)** | 104.1 ± 1.4 | 104.7 ± 1.2 | 105.4 ± 1.3 | 102.8 ± 1.2 | 104.1 ± 1.5 | 104.0 ± 1.7 | 104.7 ± 2.5 | 102.3 ± 1.8 |
| **Cholesterol**  **(mg/dL)** | 84.3 ± 17.2 | 65.8 ± 10.4 | 65.3 ± 16.1 | 78.7 ± 17.9 | 99.9 ± 15.9* | 161.7 ± 59.7* | 191.6 ± 40.9* | 202.7 ± 99.6* |
| **Creatine kinase (IU/L)** | 526.6 ± 414.1 | 479.3 ± 404.1 | 396.8 ± 322.3 | 398.7 ± 276.8 | 498.3 ± 443.2 | 531.2 ± 397.9 | 450.2 ± 466.3 | 291.8 ± 130.1 |
| **Gamma glutamyltransferase (IU/L)** | 3.0 ± 0 | 3.0 ± 0 | 3.0 ± 0 | 3.0 ± 0 | 3.0 ± 0 | 3.0 ± 0 | 3.0 ± 0 | 3.1 ± 0 |
| **Globulin**  **(g/dL)** | 1.9 ± 0.2 | 1.9 ± 0.3 | 1.9 ± 0.2 | 2.1 ± 0.2 | 1.9 ± 0.2 | 2.4 ± 0.4* | 2.6 ± 0.2* | 2.7 ± 0.3* |
| **Glucose**  **(mg/dL)** | 94.7 ± 12.3 | 108.3 ± 12.2 | 119.1 ± 14.2 | 117.3 ± 11.3 | 97.1 ± 12.8 | 110.3 ± 17.1 | 108.1 ± 17.3 | 103.9 ± 14.0* |
| **Inorganic phosphorus**  **(mg/dL)** | 8.66 ± 0.43 | 8.64 ± 0.83 | 8.17 ± 0.52 | 8.11 ± 0.41 | 8.63 ± 0.56 | 8.28 ± 0.38 | 8.14 ± 0.52 | 8.13 ± 0.55 |
| **Potassium**  **(mmol/L)** | 4.82 ± 0.28 | 4.73 ± 0.29 | 4.74 ± 0.18 | 4.84 ± 0.26 | 4.87 ± 0.28 | 5.23 ± 0.36* | 5.29 ± 0.39* | 5.08 ± 0.47 |
| **Sodium**  **(mmol/L)** | 146.1 ± 0.9 | 145.1 ± 1.0 | 146.5 ± 2.0 | 142.9 ± 1.0 | 145.4 ± 1.1 | 146.0 ± 1.5 | 145.9 ± 2.5 | 143.1 ± 1.8 |
| **Total Bilirubin (mg/dL)** | 0.10 ± 0 | 0.10 ± 0 | 0.10 ± 0 | 0.10 ± 0 | 0.10 ± 0 | 0.12 ± 0.04 | 0.14 ± 0.09 | 0.18 ± 0.11 |
| **Total Protein**  **(g/dL)** | 5.9 ± 0.26 | 5.7 ± 0.45 | 6.0 ± 0.12 | 6.3 ± 0.24 | 5.6 ± 0.3* | 5.5 ± 0.2 | 5.8 ± 0.3* | 5.9 ± 0.2* |
| **Triglycerides (mg/dL)** | 42.0 ± 21.0 | 38.8 ± 16.9 | 42.7 ± 24.2 | 49.3 ± 15.2 | 63.2 ± 20.4* | 113.4 ± 63.3* | 108.2 ± 44.3* | 141.9 ± 75.0* |

Data presented as Mean ± SD, N = 12/group.

***** Anti-Fx1A group is significantly different (p ≤ 0.05) from vehicle control group at the same time point.

**Supporting Table 4.** Urinalysis data by group and time point in vehicle control and anti-Fx1A-treated rats.

| **Treatment**  **Group** | **Vehicle** | | | | **Anti-Fx1A 7.5 mL/kg** | | | | |
| --- | --- | --- | --- | --- | --- | --- | --- | --- | --- |
| **Sample Day**  **Post-treatment** | Day 9 | Day 16 | Day 21 | Day 28 | | Day 9 | Day 16 | Day 21 | Day 28 |
| **Color**^a^ | Yellow (12) | Yellow (12) | Yellow (12) | Yellow (12) | | Yellow (12) | Yellow (12) | Yellow (12) | Yellow (12) |
| **Clarity**^a^ | Clear (12) | Clear (12) | Clear (12) | Clear (12) | | Clear (12) | Clear (12) | Clear (12) | Clear (12) |
| **Specific gravity**^b^ | 1.014 ± 0.009 | 1.013 ± 0.006 | 1.012 ± 0.008 | 1.029 ± 0.013 | | 1.015 ± 0.008 | 1.024 ± 0.007 | 1.023 ± 0.009 | 1.027 ± 0.015 |
| **pH**^b^ | 6.9 ± 0.5 | 7.0 ± 0.5 | 6.7 ± 0.3 | 6.8 ± 0.5 | | 6.9 ± 0.2 | 7.1 ± 0.2 | 7.1 ± 0.2 | 7.0 ± 0.2 |
| **Protein**^a^ | Neg (7)  Trace (3)  1+ (2) | Neg (1)  Trace (8)  1+ (3) | Neg (8)  Trace (1)  1+ (3) | Neg (1)  Trace (3)  1+ (5)  2+ (3) | | 1+ (1)  2+ (5)  3+ (6) | 3+ (12) | 3+ (12) | Neg (1)  2+ (1)  3+ (10) |
| **Glucose**^a^ | Neg (12) | Neg (12) | Neg (12) | Neg (12) | | Neg (11)  Trace (1) | Neg (12) | Neg (12) | Neg (12) |
| **Ketones**^a^ | Neg (4)  Trace (5)  1+ (3) | Trace (5)  1+ (7) | Neg (1)  Trace (1)  1+ (10) | Trace (3)  1+ (9) | | Neg (3)  Trace (7)  1+ (2) | Trace (6)  1+ (6) | Neg (1)  Trace (5)  1+ (6) | Neg (2)  Trace (7)  1+ (3) |
| **Bilirubin**^a^ | Neg (11)  1+ (1) | Neg (11)  1+ (1) | Neg (12) | Neg (9)  1+ (2)  2+ (1) | | Neg (9)  1+ (3) | Neg (11)  1+ (1) | Neg (10)  1+ (2) | Neg (10)  1+ (2) |
| **Blood**^a^ | Neg (7)  Trace (4)  2+ (1) | Neg (11)  Trace (1) | Neg (11)  2+ (1) | Neg (12) | | Neg (3)  Trace (8) | Neg (12) | Neg (11)  Trace (1) | Neg (5)  Trace (7) |
| **Urobilinogen**^a^ | [0.2](mailto:12@0.2) (12) | [0.2](mailto:12@0.2) (12) | [0.2](mailto:12@0.2) (12) | [0.2](mailto:11@0.2) (11)  [1.0](mailto:1@1.0) (1) | | [0.2](mailto:12@0.2) (12) | [0.2](mailto:12@0.2) (12) | [0.2](mailto:12@0.2) (12) | [0.2](mailto:12@0.2) (12) |
| **Urine creatinine**^b^ **(mg/dL)** | 51.7 ± 37.3 | 57.2 ± 27.3 | 57.3 ± 38.3 | 120.6 ± 56.8 | | 48.3 ± 26.3 | 82.6 ± 37.7 | 81.2 ± 31.0 | 94.3 ± 54.7 |

^a^For qualitative and semi-quantitative data findings from each animal were recorded and number of animals/group associated with each finding are indicated parenthetically, N=12/group.

^b^Data are presented as Mean ± SD, N= 12/group.

| **Analyte** | **Day** | **p Value** |
| --- | --- | --- |
| Protein | Day 9 | <0.001 |
| Protein | Day 16 | <0.001 |
| Protein | Day 21 | <0.001 |
| Protein | Day 28 | <0.001 |
| Glucose | Day 9 | 0.359 |
| Glucose | Day 16 | NA† |
| Glucose | Day 21 | NA† |
| Glucose | Day 28 | NA† |
| Ketones | Day 9 | 1.000 |
| Ketones | Day 16 | 0.713 |
| Ketones | Day 21 | 0.135 |
| Ketones | Day 28 | 0.013 |
| Bilirubin | Day 9 | 0.304 |
| Bilirubin | Day 16 | 1.000 |
| Bilirubin | Day 21 | 0.166 |
| Bilirubin | Day 28 | 0.596 |
| Blood | Day 9 | 0.102 |
| Blood | Day 16 | 0.359 |
| Blood | Day 21 | 1.000 |
| Blood | Day 28 | 0.002 |

†Insufficient variation to estimate p Value; all results from both groups were the same value.
